# A Fragmenting Hybrid Approach for Targeted Delivery of Multiple Therapeutic Agents to the Malaria Parasite

**DOI:** 10.1002/cmdc.201100002

**Published:** 2011-01-24

**Authors:** Sumit S Mahajan, Edgar Deu, Erica M W Lauterwasser, Melissa J Leyva, Jonathan A Ellman, Matthew Bogyo, Adam R Renslo

**Affiliations:** 1 Department of Pharmaceutical Chemistry, Small Molecule Discovery Center, University of CaliforniaSan Francisco, Mission Bay Campus, 1700 4th Street, San Francisco, CA 94158 (USA), Fax: +(1) 415-514-4507; 2 Department of Pathology, Stanford School of Medicine300 Pasteur Drive, Stanford, CA 94305 (USA), Fax. (+1) 650-725-7424; 3 Department of Chemistry, University of CaliforniaBerkeley, Berkeley, CA 94720-1460 (USA), Fax: +(1)510-642-8369

**Keywords:** antiparasitic agents, drug delivery, hybrid drugs, malaria, prodrugs

Artemisinin combination therapies (ACT) represent the current standard of care in the treatment of uncomplicated malaria. The widespread adoption of ACT has been motivated by a desire to minimize the emergence of drug resistance and to address the problem of recrudescence associated with artemisinin monotherapy.^1^–^4^ We set out to explore a single-molecule ‘fragmenting hybrid’ strategy in which an artemisinin-like peroxide is employed to deliver a partner drug, only upon activation by ferrous iron in the parasite. In principle, iron(II)-dependent drug delivery from a fragmenting hybrid could alleviate unwanted off-target bioactivities of the partner drug, which would be inactive in its hybrid form.

Our design for fragmenting hybrids was inspired by earlier work demonstrating that antimalarial 1,2,4-trioxolanes, including the investigational agent arterolane (**1**, Figure 1),^5^ undergo iron(II)-promoted ring opening to afford both reactive carbon-centered radical species—the presumed agent responsible for parasite toxicity—and carbonyl containing byproducts (e.g., **5**, Scheme 1 a).^6^–^8^ In parasites, scission of the trioxolane ring is thought to be initiated by free heme, a byproduct of hemoglobin degradation in the parasite digestive vacuole (DV). Because heme concentrations are enormous (estimated at 0.4 m)^9^ in the parasite DV and significantly lower (∼10^−16^ m)^10^ in human plasma, we reasoned that the presence of this reactive species in parasites could be exploited for selective drug delivery. Thus, to achieve parasite-targeted hybrid fragmentation, we embedded a masked retro-Michael linker within the 1,2,4-trioxolane ring system (Figure 1). In the hypothesized activation–release sequence, free ferrous iron heme in the parasite DV mediates opening of the trioxolane ring, which unmasks the carbonyl function of the retro-Michael linker (**6**), leading to release of the second drug species via a β-elimination reaction (Scheme 1 b). The release of free partner drug from the linker distinguishes this approach from conventional covalent hybrids,^11^ while the introduction of the retro-Michael linker greatly expands the scope of possible conjugation chemistry as compared to other peroxidic prodrugs that require drug conjugation at a carbonyl function.^12^–^14^

**Figure 1:**
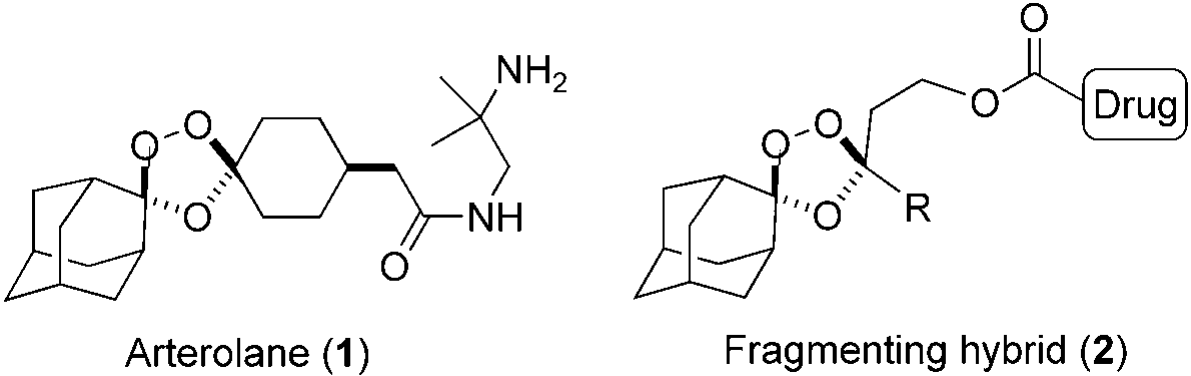
Structure of the investigational antimalarial arterolane (**1**) and an iron(II)-targeted fragmenting hybrid (**2**).

**Scheme 1:**
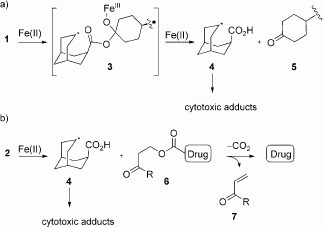
a) Mechanism of iron(II)-promoted breakdown of the 1,2,4-trioxolane ring in arterolane (**1**). b) Proposed unraveling of a fragmenting hybrid (**2**) to release an amine-bearing drug species via β-elimination from a retro-Michael substrate (**6**).

Conclusive demonstration of dual action for a hybrid drug species is challenging since simple potency comparisons are often ambiguous and difficult to interpret. Thus, although fragmenting hybrids of mefloquine and primaquine could be readily prepared, it was not trivial to establish that the quinoline species had been released from these hybrids, since the trioxolane moiety itself is a potent antimalarial agent. Therefore, we turned to studies of fragmenting hybrids containing a protease inhibitor, reasoning that delivery of such a species could be confirmed using activity-based probes of protease activity. Dipeptidyl aminopeptidase 1 (DPAP1)^15^ is an essential cysteine protease involved in the latter stages of hemoglobin degradation in the parasite DV. Inhibitors of this *exo*-protease are well suited for study of fragmenting hybrids since relevant activity-based probes are available and inhibitor potency is dramatically attenuated when the N-terminal amino group is acylated or otherwise blocked.^16^, ^17^ Herein, we demonstrate proof of principle for the fragmenting hybrid approach by demonstrating the successful release of the potent and irreversible DPAP1 inhibitor ML4118S^15^ from fragmenting hybrid **8** (Figure 2). We anticipated that ML4118S in its hybrid form **8** should possess very weak DPAP1 inhibitory activity since the linkage is made at the terminal amino function. Therefore, the observation of DPAP1 inhibition by hybrid **8** in parasites would indicate successful delivery of active ML4118S from the hybrid. We could measure the inhibitory activity in parasite extracts using FY01 (Figure 3 a), a fluorescently labeled activity-based probe that specifically targets DPAPs.^18^ Although not a viable drug candidate for various reasons, hybrid **8** has proven to be a useful tool for validating the fragmenting hybrid concept in live parasites.

**Figure 2:**
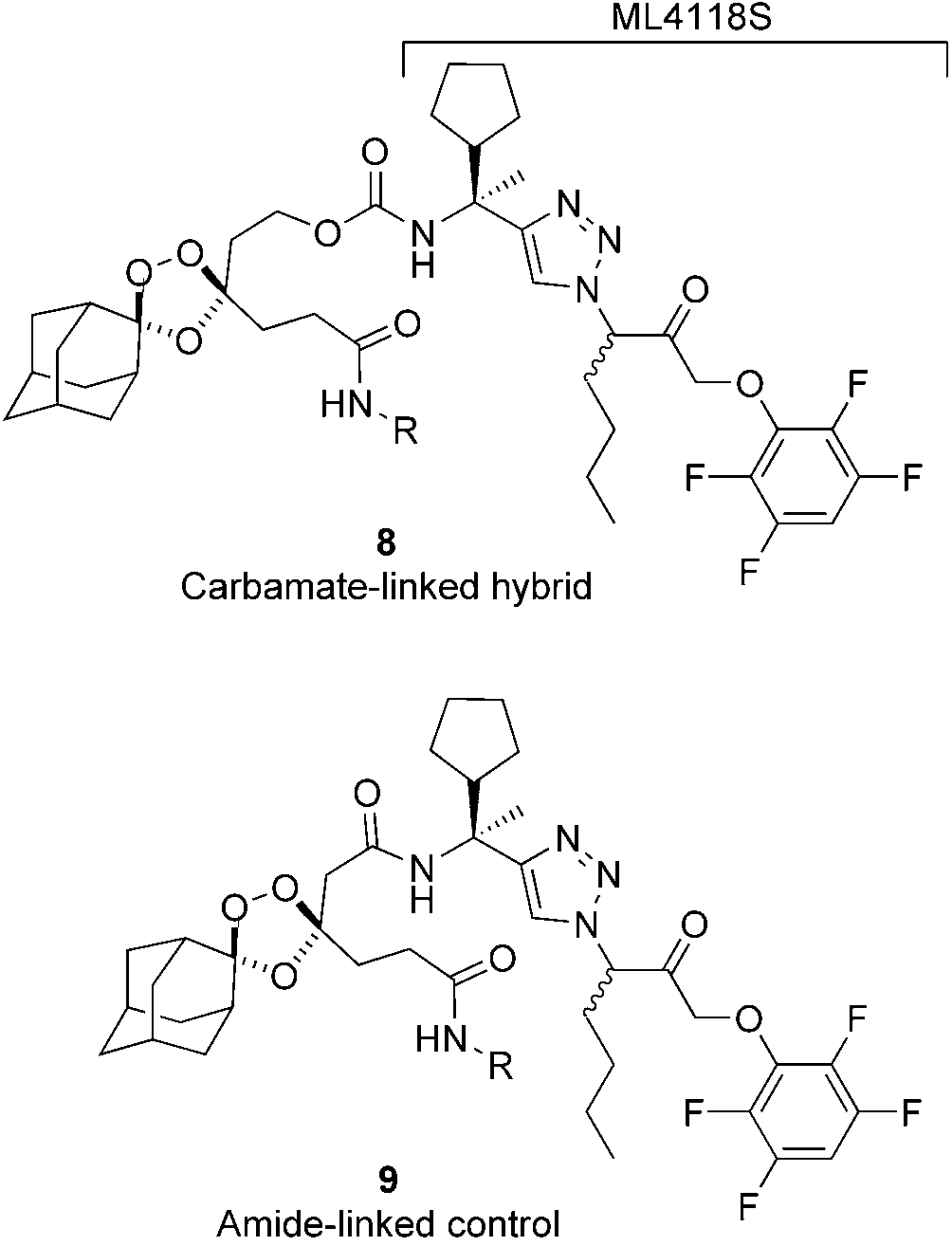
Structures of the carbamate-linked fragmenting hybrid **8** and an amide-linked congener **9**. Compound **9** is an important control compound that can be activated by ferrous iron but cannot release free ML4118S. Both **8** and **9** contain the irreversible DPAP1 inhibitor ML4118S, the α-keto position of which is not configurationally stable.^15^ R= CH_2_CH_2_CH_2_-*N*-pyrrolidinone.

**Figure 3:**
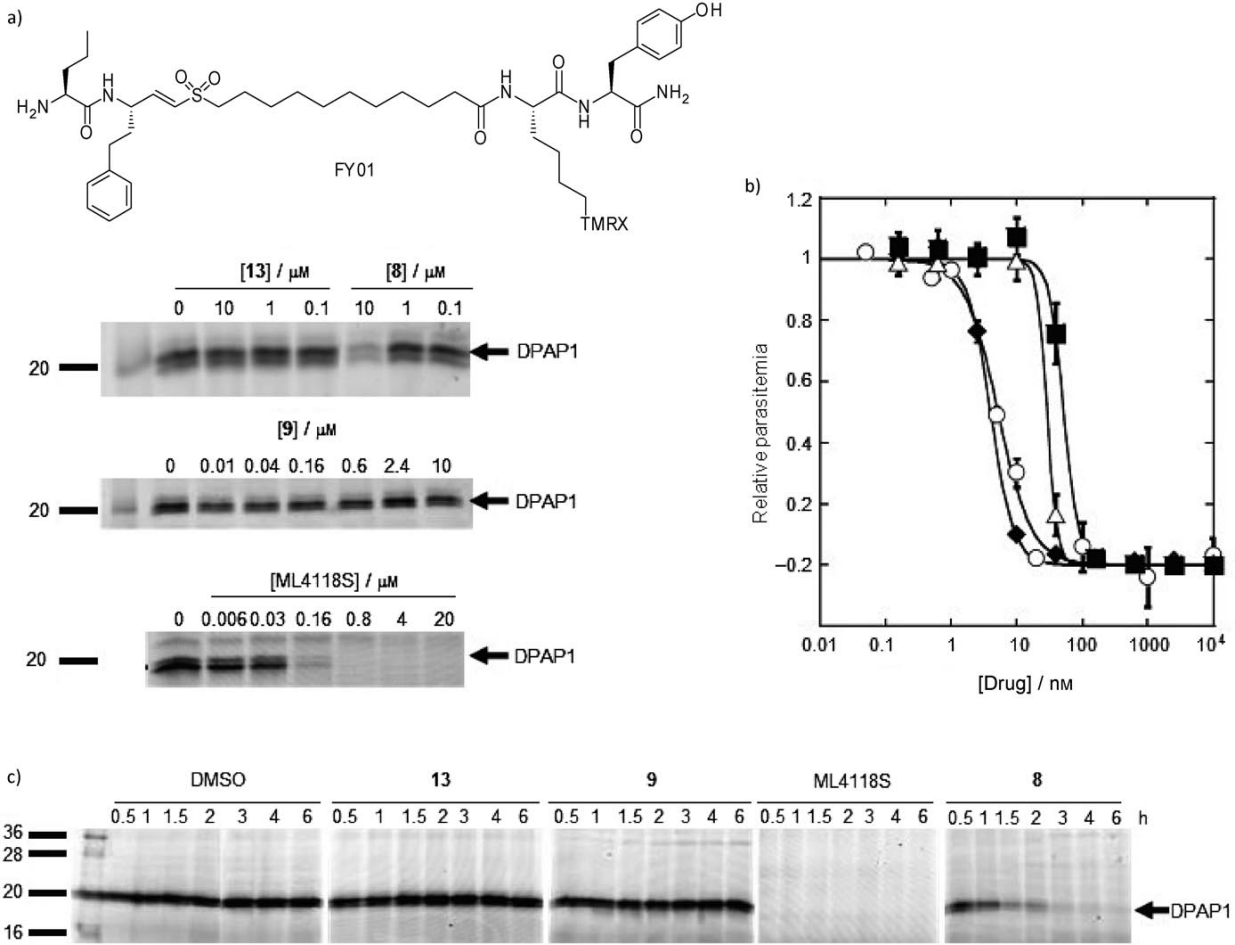
Validation of the fragmenting hybrid concept in live parasites. a) Activities of ML4118S and **8**, **9**, **13** towards DPAP1. Parasite lysates were treated for 30 min in acetate buffer with different concentrations of hybrid **8**, trioxolane **13**, ML4118S, or the control hybrid **9**. Residual DPAP1 activity was labeled with 1 μm of FY01 for 1 h and visualized by fluorescent scan of SDS-PAGE gels. b) Potency against *P. falciparum* parasites in culture: ML4118S (○); **8** (⧫); **9** (▪); **13** (▵). Ring stage parasites were treated with increasing concentrations of the indicated compound and cultured for ∼75 h. Parasitemia was quantified by FACS analysis and fitted to a dose–response curve. The EC_50,Pot_ values are reported in Table 1. c) Kinetics of DPAP1 inhibition in vivo. A synchronous culture of *P. falciparum* at trophozoite stage was treated with 50 nm of the indicated compound or DMSO. After 0.5–6 h of treatment, parasites were separated from the erythrocytes by saponin lysis, and the residual DPAP1 activity was labeled with 1 μm of FY01 in acetate buffer containing 1 % nonidet P40.

Compounds **8** and **9** were prepared from the key trioxolane intermediate **13**, which was in turn prepared using established methods of trioxolane synthesis.^19^, ^20^ Briefly, Griesbaum co-ozonolysis of 2-adamantanone methyl oxime and cyclohexa-1,4-dione afforded ketone **11**, which following Baeyer–Villiger oxidation provided lactone **12** on a gram scale (Scheme 2). Ring opening of **12** with a primary amine provided alcohol **13**, and this material could then be joined to ML4118S via a carbamate linkage to provide hybrid **8** in 38 % yield. Similar coupling reactions with the more reactive and less hindered amines present in the antimalarial drugs primaquine and mefloquine proceeded in yields of >80 % and >55 %, respectively (unpublished results). The ability to form fragmenting hybrids from a variety of primary and secondary amines is significant because most of the agents currently used in malaria combination therapies possess such functional handles. Also prepared from **13** was the crucial amide-linked control compound **9**, which can be activated by ferrous iron but cannot release free ML4118S because β-elimination is precluded. Hence, two-step oxidation of alcohol **13** to the corresponding carboxylic acid (74 % overall yield), followed by HATU-mediated coupling to ML4118S, afforded conjugate **9** in 48 % yield.

**Scheme 2:**
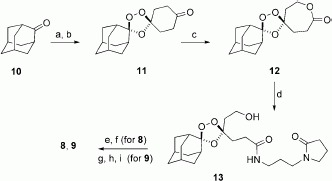
Synthesis of fragmenting hybrid **8** and nonfragmenting control compound **9**. *Reagents and conditions*: a) MeONH_2_, pyridine, RT, 48 h, 83 %; b) O_3_, cyclohexa-1,4-dione, pentane/CH_2_Cl_2_ (3:2), 0 ^o^C, 1 h, 33 %; c) *m*CPBA, NaHCO_3_, CH_2_Cl_2_, RT, 48 h, 56 %; d) *N*-(3-aminopropyl)-2-pyrrolidinone, toluene, 50 ^o^C, 5 h, 64 %; e) *p*-NO_2_PhOC(O)Cl, Et_3_N, DMAP, CH_2_Cl_2_, RT, 16 h, 96 %; f) ML4118S, DMF, DMAP, RT, 16 h, 38 %; g) Dess–Martin periodinane, CH_2_Cl_2_, RT, 30 min; h) 1 m KMnO_4_, 5 % NaH_2_PO_4_, *t*BuOH, RT, 30 min, 74 % (two steps); i) ML4118S, HATU, HOBt, DMF, DIEA, RT, 2 h, 48 %. Abbreviations: 2-(7-Aza-1*H*-benzotriazole-1-yl)-1,1,3,3-tetramethyluronium hexafluorophosphate (HATU); *meta-*Chloroperoxybenzoic acid (*m*CPBA); *N*,*N*-Diisopropylethylamine (DIEA); 4-Dimethylaminopyridine (DMAP); *N*,*N*-Dimethylformamide (DMF); 1-Hydroxybenzotriazole (HOBt).

With hybrid **8** in hand, validation of the proposed iron(II)-promoted fragmentation chemistry was undertaken, employing LC/MS for the detection of reaction products (Scheme 1). Using reaction conditions that are standard in the field,^21^ 0.3 mmol of hybrid **8** was treated with 100 equivalents of ferrous bromide in a 1:1 mixture of water and acetonitrile. These conditions afforded rapid fragmentation of the trioxolane ring and clean formation of the expected products: lactone **14** and retro-Michael substrate **15** (Scheme 3). The subsequent β-elimination reaction of **15** to generate **16** and ML4118S was also observed and was slower than the initial reaction with iron (see Supporting Information). In control experiments without ferrous bromide, hybrid **8** remained intact throughout the experimental time period. These results thus confirm that trioxolane scission and hybrid fragmentation proceeds as predicted, effecting iron-dependent release of ML4118S.

**Scheme 3:**
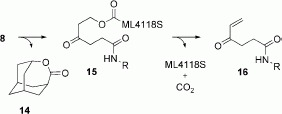
Observed reaction products (LC/MS) following treatment of fragmenting hybrid **8** with excess ferrous bromide. R= CH_2_CH_2_CH_2_-*N*-pyrrolidinone.

With the fragmentation chemistry validated in vitro, we turned to the study of **8**, **9**, **13**, and ML4118S in the context of cultured intra-erythrocytic *Plasmodium falciparum* parasites. We first measured inhibition of DPAP1 by treating parasite lysates for 30 min with increasing concentrations of test compounds followed by labeling of residual DPAP1 activity with FY01 (Figure 3 a). Free ML4118S fully inhibited DPAP1 with an IC_50_ value of 70 nm. As expected, neither trioxolane **13** alone nor the hybrid control **9** blocked DPAP1 activity, even at the highest concentration studied (10 μm). Thus, the DPAP1 activity of ML4118S is blocked in the hybrid form (**9**). Hybrid **8** partially inhibited DPAP1 at the highest concentration (10 μm), but was at least 100-fold less potent than ML4118S itself, presumably due to capping of its free amine function in the hybrid form (Table 1). An authentic sample of the linker side product **16** (see scheme S1 in the Supporting Information for the synthesis) had no effect on parasite viability or DPAP1 inhibition (Table 1; figure S3 in the Supporting Information).

**Table 1:** DPAP1 Inhibitory activities, antimalarial activities, and rates of hybrid fragmentation in vitro and in parasite cultures.^[a]^

Compd	IC_50_^[b]^ [nm]	EC_50,Pot_^[c]^ [nm]	*t*_1/2_ [h]
	DPAP1	*P. falciparum*	in vitro^[d]^	in vivo^[e]^
ML4118S	70 (13)	5.2 (0.4)	n/a	n/a
**8**	10 000	4.0 (0.2)	9	1.5 (0.25)
**9**	>10 000	52 (7)	n/a	n/a
**13**	>10 000	29 (13)	n/a	n/a
**16**	>10 000	>10 000	n/a	n/a

[a] n/a: not applicable. The standard deviation for measured values is shown in parentheses. [b] Half maximal inhibition of DPAP1 in parasite lysates after 30 min treatment with inhibitor. DPAP1 activity was measured using the FY01 probe. [c] Antimalarial potency measured by treating a culture of *P. falciparum* at ring stage with increasing concentrations of compound. The decrease in parasitemia was quantified by FACS analysis and fitted to a dose–response curve. [d] Half-life for the release of ML4118S from hybrid species **8** as measured in vitro by LC/MS spectrometry. [e] Half-life for the release of ML4118S from hybrid **8** in living parasites. This value was estimated based on the kinetics of DPAP1 inhibition observed in culture and the independently determined second-order rate constant for inhibition of DPAP1 by ML4118S in vitro.

We next evaluated the antimalarial activities of the compounds using intra-erythrocytic *P. falciparum* parasites (Figure 3 b and Table 1). Trioxolane intermediate **13** and hybrid control **9** exhibited antimalarial activities in the mid-nanomolar range (EC_50,Pot_=29 nm and 52 nm, respectively). The potent activity of **9** confirms action via the trioxolane moiety, since no inhibition of DPAP1 is conferred by this compound in live parasites (see below). The observation of trioxolane-based activity in **9** also rules out an alternative decomposition mechanism involving acid-mediated Hock fragmentation. Hock fragmentation would not produce cytotoxic carbon radicals, and thus the observation of potent activity by **9** (and **13**) suggests action via the canonical mechanism of trioxolane toxicity (Scheme 1). Significantly, ML4118S and its hybrid form **8** were both active at single-digit nanomolar concentrations (EC_50,Pot_=5.2 nm and 4.0 nm, respectively), approximately tenfold more potent than **9** or **13**. The enhanced potency of **8** relative to **9** suggests that active ML4118S is indeed released from hybrid **8** within parasites; that hybrid **8** and ML4118S have similar potencies suggests additive activity rather than synergistic or antagonistic activity.

To further investigate the release of ML4118S from hybrid **8** in parasites, we measured the kinetics of DPAP1 inhibition using the FY01 probe (Figure 3 c). No inhibition of DPAP1 activity was observed upon exposure of parasites to compound **13** for 0.5–6 h, which indicates that the toxic effects of the trioxolane moiety do not alter the levels of DPAP1 activity for at least 6 h. As expected, ML4118S completely inhibited DPAP1 activity in parasites at all time points while control hybrid **9** was unable to inhibit DPAP1 even after 6 h. Hybrid **8**, on the other hand, inhibited DPAP1 in a time-dependent fashion, with complete inhibition observed after 3 h. These results are consistent with a slow release of ML4118S from fragmenting hybrid **8**. Based on the inhibition rate constant measured for ML4118S in vitro (*k*_i_=10 200 m^−1^ s^−1^; figure S2 a in the Supporting Information), we estimate the *t*_1/2_ of ML4118S release from hybrid **8** in parasites to be ∼1.5 h (*k*_r_=0.00013 s^−1^; figure S2 b in the Supporting Information), which is sufficiently rapid to be useful in the context of antimalarial therapy.

In summary, we have shown that a prototypical fragmenting hybrid delivers multiple antimalarial activities in a targeted fashion to the intra-erythrocytic *P. falciparum* parasite. In principle, the slow release of a partner drug in such hybrids could complement the rapid action of the trioxolane moiety, much as the ACT strategies seek to combine a longer-acting partner drug with a rapid-acting artemisinin. As demonstrated with hybrid **8**, intrinsic bioactivities of the partner drug can be masked in the hybrid form, raising the possibility that undesired on/off-target effects of known drugs might similarly be attenuated using this approach. Antimalarial agents like primaquine and amodiaquine that exhibit systemic toxicities might be more safely administered in the form of a fragmenting hybrid, as this would limit systemic exposure to free partner drug. More speculatively, agents conferring irreversible target inhibition or polypharmacology might be safely delivered using a fragmenting hybrid approach in which systemic exposure to free drug is avoided. Currently, we are exploring next-generation fragmenting hybrids that overcome limitations of the initial prototypical systems described herein.

## Notes

Detailed facts of importance to specialist readers are published as ”Supporting Information”. Such documents are peer-reviewed, but not copy-edited or typeset. They are made available as submitted by the authors.
