# Supplementary material for: A Fragmenting Hybrid Approach for Targeted Delivery of Multiple Therapeutic Agents to the Malaria Parasite
Source: ChemMedChem. 2011 Jan 24;6(3):415–9. doi: 10.1002/cmdc.201100002 (PMC3265971; doi:10.1002/cmdc.201100002)
Supplement: Supplementary file 1 [file cmdc0006-0415-sd1.pdf]

CHEMMEDCHEM Chemistry & Drug Discovery

Supporting Information

© Copyright Wiley-VCH Verlag GmbH & Co. KGaA, 69451 Weinheim, 2011

A Fragmenting Hybrid Approach for Targeted Delivery of Multiple Therapeutic Agents to the Malaria Parasite

Sumit S. Mahajan,[a] Edgar Deu,[b] Erica M. W. Lauterwasser,[a] Melissa J. Leyva,[c] Jonathan A. Ellman,[c] Matthew Bogyo,*[b] and Adam R. Renslo*[a]

cmdc_201100002_sm_miscellaneous_information.pdf

Supplementary Information

Table of Contents

1) Supplementary Figures and Schemes
2) Experimental Procedures – Kinetic Studies
3) Experimental Procedures – Chemical Biology
4) Experimental Procedures – Chemical Synthesis

1) Supplementary Figures

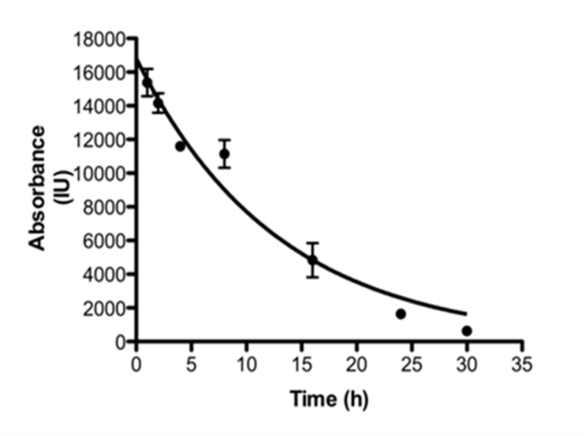

Supplementary Figure 1. Kinetics of *in vitro* fragmentation of hybrid **8** at 37 °C in the presence of $FeBr_2$. Shown is the rate-determining disappearance of the retro-Michael intermediate **15** (Scheme 3) as followed by LC/MS analysis.

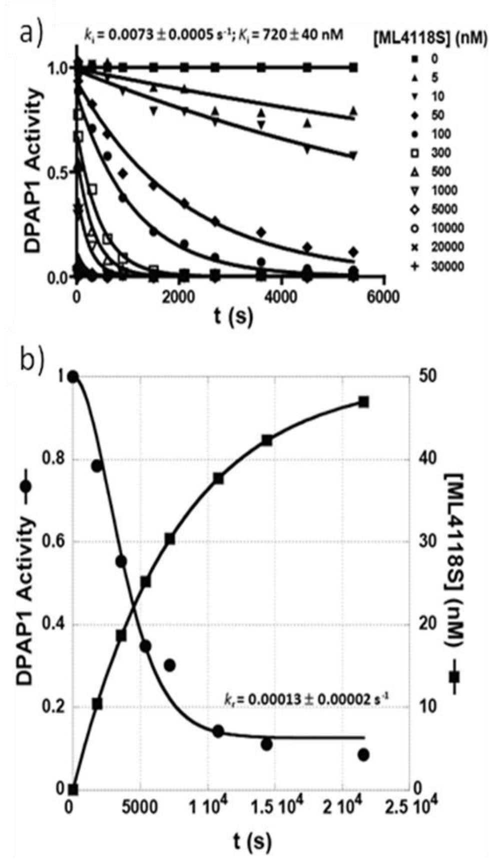

Supplementary Figure 2. Measurement of the rate of hybrid **8** decomposition *in vivo*. a) Rate of DPAP1 inhibition by **ML4118S** *in vitro*. Parasite lysates were treated for 15 to 90 min with a dilution series of inhibitor. The residual activity for each **ML4118S** concentration and treatment time was measured as the turnover rate of 10 μM of $(Pro\text{-}Arg)_2$-Rho over 5 min. The fraction of DPAP1 activity relative to the DMSO controls was fitted to eq 2. The inhibition parameters thus obtained are shown. b) Estimation of the rate of release of **ML4118S** from hybrid **8** in living parasites. DPAP1 activity was quantified from the gel shown in Figure 2c and normalized based on the DMSO control values (circles). These values were fitted to eq 2 using the k_i and K_i values shown in part a of this figure and replacing [I] by the expression of [**ML4118S**] in eq 4. The rate of release (k_r) thus obtained is reported in the figure. Square symbol simulate the concentration of **ML4118S** that is released inside the food vacuole over time (eq 4). After 1.5 h of treatment the concentration of **ML4118S** is expected to have reached half of the initial concentration of hybrid **8** (i.e. 25 nM).

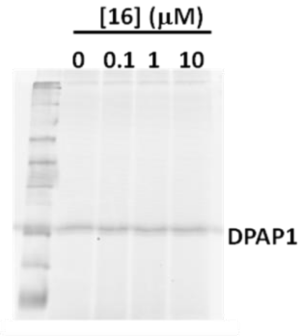

Supplementary Figure 3. Activity of linker side product **16** towards DPAP1. Parasite lysates were treated for 30 min in acetate buffer with different concentrations of **16**. Residual DPAP1 activity was labeled with 1 μM of **FY01** for 1 h, and visualized by fluorescent scan of SDS-PAGE gels. No significant inhibition of DPAP1 by **16** is observed in this experiment.

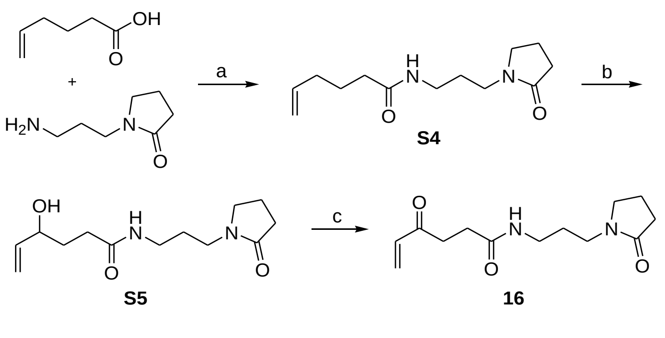

Supplementary Scheme 1. Synthesis of an authentic sample of retro-Michael linker side product **16**. Conditions: (a) HATU, DIEA, DMF, 95%; (b) SeO_2, *t*-BuOOH, CH_2Cl_2, 67%; (c) Dess-Martin periodinane, CH_2Cl_2, 61%.

2) Experimental Procedures – Kinetic Studies

Study of Iron(II)-promoted breakdown of 8 *in vitro*. A solution of hybrid **8** (0.3 mM) in 1 ml of 1:1 CH_3CN:H_2O was treated with $FeBr_2$ (30 mM) and stirred at 37 ^oC. Aliquots were taken at various time points and analyzed by LC/MS (Waters Micromass ZQTM/Waters 2795 Separation Module/Waters 2996 Photodiode Array Detector/XTerra® MS C18, 5µm, 4.6 x 50 mm column). The LC method involved gradient elution from 0-95% CH_3CN in water (constant 0.2% formic acid) over 12 min. The rate-determining β-elimination reaction was followed by observing the decreasing absorbance (λ = 254 nM) of the peak corresponding to the retro-Michael substrate **15** (Scheme 3). This signal was integrated at various time points using Mass Lynx software. Graphpad prism was used to fit the data to a single exponential decay equation and to derive a half-life ($t_{½}$) for the elimination reaction of ~9 hours (Supplementary Figure 1).

Determination of ML4118S K_i using a fluorogenic activity assay for DPAP1. Trophozoite lysates in acetate buffer (1 to 10 dilution) were treated with increasing concentrations of **ML4118S** for 15 to 90 min. Residual DPAP1 activity was measured as the turnover rate of $(Pro\text{-}Arg)_2$-Rho, a DPAP1 specific substrate[1] in parasite lysates, by diluting the treated samples 10-fold in acetate buffer (50 mM sodium acetate pH 5.5, 5 mM $MgCl_2$, and 5 mM DTT) containing 10 µM of substrate. Substrate turnover was measured for 5 min in a 96-well plate at 523 nm (using an excitation wavelength of 492 nm and an emission cutoff filter at 515 nm) in a Spectramax M5 plate-reader (Molecular Devices).

The rates of substrate turnover relative to DMSO controls (v/v_0) were fitted to the irreversible inhibitor model shown in equation 1 with equation 2.

$$E + I \underset{}{\overset{K_i}{\rightleftharpoons}} E\,I \xrightarrow{k_i} E\text{-}I \qquad \text{(eq 1)}$$

$$\frac{v}{v_0} = \frac{\exp\left(-\frac{k_i[I]\cdot t}{K_i + [I]}\right)}{1 + [I]/K_i} \qquad \text{(eq 2)}$$

where E, I, E I, and E-I represent free enzyme, inhibitor, inhibitor bound to the enzyme, and the enzyme-inhibitor covalent complex, respectively. t is the treatment time of lysates with inhibitor, *K*i the dissociation constant of the non-covalent enzyme:inhibitor complex, and *k*i is the rate constant of covalent modification of the enzyme by the inhibitor.

Estimation of the rate of ML4118S release from hybrid 8 in living parasites. Three assumptions were taken to estimate the rate of the retro-Michael reaction for hybrid **8** *in vivo*: 1) The rate of DPAP1 inhibition by **ML4118S** inside the food vacuole is identical to that measured *in vitro* in acetate buffer. 2) The initial concentration of hybrid **8** inside the parasites is identical to the one in the media. 3) The kinetic of the β-elimination reaction can be approximated to a simple single exponential decay (eq 3).

$$\mathbf{8} \xrightarrow{fast} \mathbf{14} + \mathbf{15} \xrightarrow{kr} \mathbf{ML4118S} + \mathbf{16} \qquad \text{(eq 3)}$$

where k_r is the average rate of release of **ML4118S** in the food vacuole, which is much slower than the reaction between iron(II) and the trioxolane moiety of compound **8**. The formation of **ML4118S** can therefore be described by (eq 4).

$$[\mathrm{ML4118}S] = [8]_0\left(1 - \exp(-k_r t)\right) \qquad \text{(eq 4)}$$

where k_r is the average rate of release of **ML4118S** in the food vacuole, and $[\mathbf{8}]_0$ is the initial concentration of hybrid **8**, i.e. 50 nM. To obtain k_r, the residual DPAP1 activity values, measured during the treatment of a parasite culture with 50 nM of hybrid **8** (Figure 3c), were fitted to eq 2 where [I] was replace by [**ML4118S**] as it is defined in eq 4, and the k_i and K_i values were fixed to those determined *in vitro* for **ML4118S** (Supplementary Fig 2a).

3) Experimental Procedures – Chemical Biology

Parasite culture, harvesting and lysate preparation. D10 *P. falciparum* clones were cultured with media containing Albumax (Invitrogen) using standard procedures[2, 3]. D10 parasites were synchronized every 48 h at ring stage by treatment with 5 % sorbitol. Parasite pellets were harvested at trophozoite stage by selectively lysing the RBC membranes with 0.15 % saponin (Calbiochem, San Diego, CA). Lysates were prepared by treating one volume of parasite pellet with two volumes of 1 % nonidet P40 in PBS for 1 h on ice. The soluble fraction was separated from the insoluble one by a 5 min microcentrifugation at 13000 rpm.

***P. falciparum* replication assay.** 200 µL of synchronized cultures of D10 parasites (~ 2 % parasitemia and 0.5 % hematocrit) were treated at ring stage with increasing concentrations of compound and were left to grow in 96-well plates for ~75 h. Cells were fixed in 0.05 % glutaraldehyde (Sigma) in PBS for at least 12 h at 4 °C, permeabilized for 5 min with 0.25 % Triton X in PBS, and stained with 0.05 mg/mL of propidium iodide (Sigma) in water. Infected and uninfected RBCs were quantified by FACS as the populations with positive and negative fluorescence in the propidium iodide channel, respectively.[4] All FACS measurements were taken on a BD FACScan flow cytometer (Becton, Dickinson and Co.). All $EC_{50,Pot}$ values for parasite death were obtained by fitting the percentage parasitemia to a dose response curve.

Labeling of DPAP1 activity with FY01. **FY01** is a cell permeable BODIPY-TMR fluorescently-tagged probe containing a vinyl sulfone reactive group that covalently modifies the catalytic cysteine of DPAP1.[4] Parasite lysates were diluted 10-times in acetate buffer and treated with 1 µM **FY01** for 1 h at room temperature. To measure the specificity of an inhibitor against DPAP1, parasite lysates were treated with increasing concentrations of inhibitor for 30 min prior to labeling with **FY01**. Samples were then boiled in SDS-loading buffer and run on a SDS-PAGE gel. DPAP1 labeled bands run as a doublet around 20 kDa and were directly detected in a 9410 Typhoon Scanner (Amersham Bioscience, GE Healthcare).

Kinetics of DPAP1 inhibition in living parasites. A synchronous culture of parasites (~ 20 % parasitemia) at trophozoite stage was cultured with 50 nM of compounds **8**, **9**, **13**, **ML4118S** or DMSO. 1mL aliquots of culture were taken after 0.5 to 6 h of treatment, and the RBC membranes were lysed with 0.15 % saponin. Parasite pellets were resuspended in acetate buffer containing 1% nonidet

P40, and DPAP1 activity was labeled with 1 µM FY01 at room temperature for 1 h.

4) Experimental Procedures – Chemical Synthesis

General Methods

^{1}H NMR spectra were recorded on a Varian INOVA-400 400 MHz spectrometer. Chemical shifts are reported in δ units (ppm) relative to TMS as an internal standard. Coupling constants (*J*) are reported in hertz (Hz). All reagents and solvents were purchased from Aldrich Chemical or Acros Organics and used as received unless otherwise indicated. Some synthetic intermediates were synthesized according to literature methods as indicated. Air and/or moisture sensitive reactions were carried out under an argon atmosphere in oven-dried glassware using anhydrous solvents from commercial suppliers. Air and/or moisture sensitive reagents were transferred via syringe or cannula and were introduced into reaction vessels through rubber septa. Solvent removal was accomplished with a rotary evaporator at ca. 10-50 Torr. Column chromatography was carried out using a Biotage SP1 flash chromatography system and silica gel cartridges from Biotage. Analytical TLC plates from EM Science (Silica Gel 60 F254) were employed for TLC analyses. Mass analyses and compound purity were determined using Waters Micromass ZQTM, equipped with Waters 2795 Separation Module and Waters 2996 Photodiode Array Detector. Separations were carried out with an XTerra® MS C18, 5µm, 4.6x50 mm column, at ambient temperature (unregulated) using a mobile phase of water-acetonitrile containing a constant 0.20 % formic acid.

Preparation of Adamantane-2-spiro-3'-9'-oxo-1',2',4',8'-tetraoxaspiro[4.6]undecane (12). To a solution of adamantane-2-spiro-3'-8'-oxo-1',2',4'-trioxaspriro[4.5]decane (**11**, 1.0 g, 3.6 mmol), prepared as described previously[5] in dichloromethane (60 ml) was added solid $NaHCO_3$ (0.6 g, 7.2 mmol), and *m*-chloroperbenzoic acid (1.8 g, 7.9 mmol). The white cloudy solution was stirred for two days at room temperature and then diluted with 40 ml of water, and the layers separated. The aqueous phase was extracted thrice with dichloromethane and the combined organic phases washed with dilute $NaHCO_3$, brine, and dried (Na_2SO_4), filtered, and concentrated to afford an oil that solidified on standing. This material was purified by column chromatography (0-15% EtOAc-hexanes) to afford lactone **12** (0.60 g, 2.0 mmol, 56%). ^{1}H NMR (400 MHz, $CDCl_3$) δ: 4.29 (m, 2H), 2.72 (m, 2H), 2.17 (m, 2H), 2.06 (m, 2H), 2.0-1.70 (m, 14 H). ^{13}C NMR (100 MHz, $CDCl_3$) δ: 174.8, 113.1, 107.8, 94.6, 64.1, 38.6, 36.7, 36.4, 34.9, 34.9, 34.9, 34.8, 32.1, 28.8, 26.9, 26.5. LRMS calculated for $C_{16}H_{22}O_5$ MH^+ 295.15 found 295.6.

Preparation of 3-(3-(2-hydroxyethyl)-5,5-spiroadamantyl-1,2,4-trioxolan-3-yl)-*N*-(3-(2-oxopyrrolidin-1-yl)propyl)propanamide (13). A solution of adamantane-2-spiro-3'-9'-oxo-1',2',4',8'-tetraoxaspiro[4.6]undecane (**12**) (160 mg, 0.55 mmol) was dissolved in the minimum amount of toluene. To this solution was added 1-(3-aminopropyl)-2-pyrrolidinone (0.15 ml, 1.11 mmol) and the reaction mixture was stirred at 50 ^{o}C for 5 hr. The solvent was evaporated and the residue was dissolved in CH_2Cl_2 (10 ml). The organic solution was washed with water, dried (Na_2SO_4), filtered, and evaporated to afford an oil. The crude material was purified by silica gel chromatography (0-10% MeOH-CH_2Cl_2) to afford alcohol **13** (153 mg, 0.35 mmol, 64% yield) as a colorless oil. ^{1}H NMR (400 MHz, $CDCl_3$) δ: 6.87 (t, *J* = 5.6 Hz, 1H), 3.76 (s, 2H), 3.36 (m, 2H), 3.30 (t, *J* = 6.4 Hz, 2H), 3.15 (m, 2H), 3.01 (s, 1H), 2.38-1.60 (m, 26 H); ^{13}C NMR (100 MHz, $CDCl_3$) δ: 176.2, 172.6, 112.4, 110.92, 58.66, 47.56, 39.75, 37.92, 36.83, 36.45, 35.72, 35.16, 34.97, 31.84, 31.31, 31.13, 26.92, 26.54, 18.09. LRMS calculated for $C_{23}H_{36}N_2O_6$ MH^+ 436.54 found 437.4.

Preparation of the nitrophenylcarbamate of 13 (S1). To a solution of alcohol **13** (100 mg, 0.23 mmol) in anhydrous CH_2Cl_2 (3 mL) was added triethylamine (0.06 mL, 0.46 mmol), 4-nitrophenyl chloroformate (92 mg, 0.46 mmol), and 4-dimethylaminopyridine (28 mg, 0.23 mmol). The solution was stirred for 16 h at rt. The reaction was diluted with CH_2Cl_2 (10 mL), washed with a saturated aqueous solution of sodium bicarbonate (3 x 5 mL), water (10 mL), and brine; the organic layer was dried over Na_2SO_4 and concentrated *in vacuo*. The product was purified from the crude residue by flash silica column chromatography, eluting with 4% methanol in CH_2Cl_2 (135 mg, 0.22 mmol, 96%): IR 3305, 2931, 2858, 1768, 1666, 1616, 1594, 1525, 1493, 1452, 1349, 1259, 1215, 1164, 1112, 1086, 1018 cm^{-1}; ^{1}H NMR (400 MHz, $CDCl_3$) δ 1.64-2.31 (m, 22H), 2.35 (t, *J* = 7.8, 2H), 2.41 (t, *J* = 8.6, 2H), 3.18 (q, *J* = 6.8, 2H), 3.32-3.40 (m, 2H), 3.38 (t, *J* = 7.0, 2H), 4.43 (t, *J* = 6.8, 2H), 6.78 (m, 1H), 7.39 (app. d, *J* = 8.8, 2H), 8.27 (app. d, *J* = 8.8, 2H); ^{13}C NMR (100 MHz, $CDCl_3$) δ 176.1, 171.9, 155.7, 152.5, 145.5, 125.4, 122.0, 112.7, 109.2, 65.3, 47.5, 39.5, 36.8, 36.5, 36.4, 35.5, 35.2, 35.0, 35.0, 34.8, 34.7, 31.2, 31.0, 30.8, 26.9, 26.5, 26.5, 18.1; LRMS calculated for $C_{30}H_{39}N_3O_{10}$ 601.64, found $(M+H^+)$ 602.7.

Synthesis of Fragmenting Hybrid 8. The nitrophenylcarbamate intermediate **S1** (20 mg, 0.032 mmol) in anhydrous dimethylformamide (0.3 ml) was treated with **ML4118S**, prepared as described[6], (10 mg, 0.018), diisopropylethylamine (8 µl, 0.042 mmol) and 4-dimethylaminopyridine (0.5 mg, 0.004 mmol). The reaction mixture was stirred overnight under argon and then diluted with EtOAc (10 ml) and washed with saturated $NaHCO_3$ (10 ml) and brine (10 ml). The organic layer was dried (Na_2SO_4), filtered, and evaporated. The residue obtained was purified using silica gel chromatography (0-5% MeOH-EtOAc) to afford hybrid **8** (6 mg, 0.007 mmol, 38%). ^{1}H NMR (400 MHz, $CDCl_3$) δ: 7.63-7.65 (m, 1H), 6.78-6.85 (m, 2H), 5.77 (bs, 0.4H), 5.53-5.55 (m, 1H), 4.81-4.92 (m, 2H), 4.04-4.14 (m, 2H), 3.39 (t, *J* = 7.0, 2H), 3.34 (t, *J* = 6.2, 2H), 3.18-3.19 (m, 2H), 2.69-2.70 (m, 0.6H), 2.40 (t, *J* = 7.8, 2H), 1.15-2.32 (m, 40H), 0.85-0.89 (m, 3H); ^{13}C NMR (100 MHz, $CDCl_3$) δ: 199.6, 176.0, 172.5, 152.6, 121.6, 112.3, 109.8, 100.5, 100.2, 100.0, 75.4, 65.8, 60.2, 56.3, 47.5, 41.1, 39.7, 36.8, 36.4, 36.3, 35.8, 35.1, 34.9, 34.8, 34.7, 31.7, 31.2, 31.0, 30.8, 28.6, 27.8, 27.7, 27.3, 26.9, 26.6, 26.5, 25.9, 25.8, 25.8, 22.6, 22.0, 18.0, 13.8; ^{19}F NMR, ($CDCl_3$) δ: -157.490 (m, 2F), -139.443 (m, 2F). LRMS calculated for $C_{46}H_{62}F_4N_6O_9$ MH^+ 919.01 found 919.23.

Preparation of 3-(3-(2-oxoethyl)-5,5-spiroadamantyl-1,2,4-trioxolan-3-yl)-N-(3-(2-oxopyrolidin-1-yl)propyl)propanamide (S2). A solution of **13** (200 mg, 0.46 mmol) in CH_2Cl_2 (2 ml) was treated with the Dess-Martin periodinane (291 mg, 0.68 mmol) and stirred at room temperature for 30 min. The reaction was then quenched by addition of 10 ml of 1:1 mixture of saturated aqueous $NaHCO_3$ and saturated aqueous $Na_2S_2O_3$. The mixture was stirred until organic and aqueous phases became clear. Next, the layers were separated and the aqueous phase was extracted twice with diethyl ether (10 ml). The combined organic layers were dried over Na_2SO_4, filtered and concentrated to afford aldehyde **S2** (188 mg, 0.43 mmol, 94% crude), which was used in the next reaction without further purification. ^{1}H NMR (400MHz, $CDCl_3$) δ: 9.70 (s, 1H), 6.82 (br s, 1H), 3.36-3.29 (m, 4H), 3.13 (m, 2H), 2.76 (m, 2H), 2.36-

1.63 (m, 24 H); LRMS calculated for $C_{23}H_{34}N_2O_6$ MH^+ 434.53 found 435.23.

Preparation of 2-(5,5-spiroadamantyl-3-(3-(2-oxopyrolidin-1yl-amino)-3-oxpropyl)-1,2,4-trioxolan-3-yl)acetic acid (S3). Crude aldehyde **S2** (61 mg, 0.14 mmol) was dissolved in *t*-butanol (0.6 mL) and was treated with a 5% NaH_2PO_4 solution (0.4 mL) followed by a 1 M aqueous solution of $KMnO_4$ (0.6 mL). After 30 min the reaction solution was diluted with ether (7 mL) and cooled to 0 °C. A saturated solution of Na_2SO_3 (1.4 mL) was added dropwise and the pH was brought to 3 by the addition of cold 1 M HCl. The organic layer was separated and the aqueous layer was extracted with EtOAc (2 x 10 mL). The organics were dried over Na_2SO_4 and concentrated. The residue was purified by silica gel chromatography (5% MeOH-CH_2Cl_2) to afford carboxylic acid **S3** as a white powder (50 mg, 0.11 mmol, 79%). ^{1}H NMR (400 MHz, $CDCl_3$) δ: 6.93 (br s, 1H), 3.39-3.31 (m, 4H), 3.20 (m, 2H), 2.80 (q, J_{AB} = 14.4, 2H), 2.44-1.66 (m, 24 H); ^{13}C NMR (100 MHz, $CDCl_3$) δ: 176.62, 173.06, 171.31, 113.08, 108.29, 47.75, 42.95, 40.09, 36.86, 36.46, 35.42, 35.01, 34.42, 31.31, 30.84, 30.35, 27.13, 26.57, 18.22. LRMS calculated for $C_{23}H_{34}N_2O_7$ MH^+ 450.53 found 451.6.

Preparation of Hybrid Control 9. To a solution of 2-(5,5-spiroadamantyl-3-(3-(2-oxopyrolidin-1yl-amino)-3-oxopropyl)-1,2,4-trioxolan-3-yl) acetic acid (**S3**) (12 mg, 0.026 mmol) in anhydrous dimethylformamide (0.3 ml) was added **ML4118S** (8 mg, 0.014 mmol), diisopropylethylamine (8.9 μl, 0.051 mmol) and 2-(7-aza-1H-benzotriazole-1-yl)-1,1,3,3-tetramethyluronium hexafluorophosphate (HATU, 9 mg, 0.026 mmol). The reaction was stirred for 2 hr and then diluted with EtOAc (10 ml) and washed with saturated $NaHCO_3$ (10 ml) and brine (10 ml). The organic layer was then separated, dried (Na_2SO_4), filtered and evaporated. The residue obtained was purified by silica gel chromatography (0-5% MeOH-EtOAc) to afford **9** (6 mg, 0.007 mmol, 48% yield). ^{1}H NMR (400 MHz, $CDCl_3$) δ: 7.78 (s, 0.5 H), 7.77 (s, 0.5 H), 6.87-6.68 (m, 3H), 5.53 (m, 1H), 4.88 (m, 2H), 3.37-3.30 (m, 4H), 3.20 (m, 2H), 2.50-2.25 (m, 6H), 2.20-1.14 (m, 38), 0.86 (m, 3H); ^{13}C NMR (100MHz, $CDCl_3$) δ: 199.8, 175.9, 171.9, 167.2, 152.0, 122.5, 122.3, 122.0, 113.3, 113.1, 108.9, 100.3, 100.0, 75.3, 71.3, 66.1, 56.9, 56.7, 49.3, 48.9, 47.5, 45.9, 41.2, 39.9, 39.8, 36.6, 36.3, 36.1, 35.7, 35.0, 34.8, 34.4, 34.2, 31.1, 31.0, 30.7, 30.6, 30.1, 30.0, 27.8, 27.3, 26.9, 26.9, 26.8, 26.5, 25.9, 25.7, 22.8, 22.7, 22.0, 19.0, 18.1, 13.9, 11.4. ^{19}F NMR, ($CDCl_3$) δ: -157.40 (m, 2F), -139.78 (m, 2F). LRMS calculated for $C_{45}H_{60}F_4N_6O_8$ MH^+ 888.99 found 889.7.

Preparation of N-(3-(2-oxopyrrolidin-1-yl)propyl)hex-5-enamide (S4). To a solution of hexenoic acid (400 mg, 3.5 mmol), diisopropylethylamine (0.67 mL, 3.85 mmol), and HATU (1.464 g, 3.85 mmol) in DMF (0.5 mL) was added 1-(3-aminopropyl)-2-pyrrolidinone (0.59 ml, 3.85 mmol). The reaction was stirred overnight at room temperature. The solution was diluted with EtOAc (15 mL) and washed with a saturated solution of sodium bicarbonate (1x 10 mL) and water (2 x 10 mL). The combined aqueous layers were back-extracted with CH_2Cl_2 (2 x 10 mL). The organic layers were combined, dried over $MgSO_4$, and concentrated in vacuo. The crude residue was purified via flash silica gel column chromatography (5%-10% MeOH/EtOAc) to yield the desired product as a clear oil (793 mg, 3.33 mmol, 95%). ^{1}H NMR (400 MHz, $CDCl_3$) δ: 6.71 (bs, 1 H), 5.72-5.82 (m, 1 H), 4.94-5.03 (m, 2H), 3.37 (t, *J* = 7.2, 2H), 3.33 (t, *J* = 6.0, 2H), 3.17 (q, *J* = 6.1, 2H), 2.40 (t, *J* = 8.2, 2H), 2.18 (t, *J* = 7.6, 2H), 2.00-2.10 (m, 4H), 1.62-1.77 (m, 4H); ^{13}C NMR (100MHz, $CDCl_3$) δ: 176.0, 173.1, 138.1, 115.3, 47.4, 39.5, 36.3, 35.4, 33.4, 31.0, 26.6, 25.0, 18.0. LRMS calculated for $C_{13}H_{22}N_2O_2$ 238.17, found (M+H$^+$) 239.5.

Preparation of 4-hydroxy-N-(3-(2-oxopyrrolidin-1-yl)propyl)hex-5-enamide (S5). To a solution of N-(3-(2-oxopyrrolidin-1-yl)propyl)hex-5-enamide (150 mg, 0.63 mmol) in CH_2Cl_2 (1 mL) was added *t*-butyl hydroperoxide (0.63 mL of a 5-6 M solution in decane) and selenium dioxide (140 mg, 1.26 mmol). After stirring overnight at room temperature, the reaction was not complete so more selenium dioxide was added (140 mg) and the reaction mixture was stirred another 12 h. The mixture was loaded directly on a silica gel column and purified eluting with 5%-10% MeOH/CH_2Cl_2. The final product was isolated as an oil (108 mg, 0.42 mmol, 67%). ^{1}H NMR (400 MHz, $CDCl_3$) δ: 6.95 (bs, 1 H), 5.81-5.89 (m, 1 H), 5.26 (d, *J* = 17.2, 1H), 5.09 (d, 10.4, 1H), 4.17 (bs, 1H), 3.38 (t, *J* = 7.2, 2H), 3.33 (t, *J* = 6.2, 2H), 3.15-3.20 (m, 2H), 2.29-2.45 (m, 4H), 2.01-2.08 (m, 2H), 1.87-1.92 (m, 1H), 1.75-1.82 (m, 1H), 1.64-1.70 (m, 2H); ^{13}C NMR (100MHz, $CDCl_3$) δ: 176.2, 173.9, 140.9, 114.5, 72.1, 47.5, 39.6, 35.7, 32.9, 32.3, 31.0, 26.5, 18.0. LRMS calculated for $C_{13}H_{22}N_2O_3$ 254.16, found (M+H$^+$) 255.09.

Preparation of 4-oxo-N-(3-(2-oxopyrrolidin-1-yl)propyl)hex-5-enamide (16). To a solution of 4-hydroxy-N-(3-(2-oxopyrrolidin-1-yl)propyl)hex-5-enamide (32 mg, 0.13 mmol) in CH_2Cl_2 (0.7 mL) was added Dess-Martin periodinane (64 mg, 0.15 mmol). The mixture was stirred for 30 min at room temperature. The crude material was filtered and loaded directly onto a silica gel column. The product eluted in 5% MeOH/CH_2Cl_2 and was isolated as a clear viscous oil (20 mg, 0.08 mmol, 61%). ^{1}H NMR (400 MHz, $CDCl_3$) δ: 6.78 (bs, 1 H), 6.21-6.38 (m, 2H), 5.83 (dd, *J* = 6.0, 10.4, 1H), 3.32-3.39 (m, 4H), 3.17 (q, *J* = 6.2, 2H), 2.94 (t, *J* = 6.7, 2H), 2.50 (t, *J* = 6.7, 2H), 2.39 (t, *J* = 8.1, 2H), 2.03 (quin., *J* = 7.6, 2H), 2.94 (quin., *J* = 6.2, 2H); ^{13}C NMR (100MHz, $CDCl_3$) δ: 199.5, 175.9, 172.0, 136.4, 128.5, 47.4, 39.7, 35.8, 34.8, 31.0, 30.1, 26.6, 18.0. LRMS calculated for $C_{13}H_{20}N_2O_3$ 252.15, found (M+H$^+$) 253.07.

References - Supplementary Information

[1] E. Deu, Z. Yang, F. Wang, M. Klemba, M. Bogyo PlosOne, **2010**, *5*, e11985.

[2] W. Trager, J. B. Jensen, *Science* **1976**, *193*, 673.

[3] M. J. Blackman, *Methods Cell Biol* **1994**, *45*, 213.

[4] S. Arastu-Kapur, E. L. Ponder, U. P. Fonovic, S. Yeoh, F. Yuan, M. Fonovic, M. Grainger, C. I. Phillips, J. C. Powers, M. Bogyo, *Nat Chem Biol* **2008**, *4*, 203.

[5] Y. Tang, Y. Dong, J. M. Karle, C. A. DiTusa, J. L. Vennerstrom, *J. Org. Chem.* **2004**, *69*, 6470.

[6] E. Deu, M. Leyva, V. Albrow, M. J. Rice, J. A. Ellman, M. Bogyo. *Chem Biol* **2010**, *17,* 808.
